# A multi-dimensional Sustainable Diet Index (SDI) for Ghanaian adults under transition: the RODAM Study

**DOI:** 10.1186/s12937-024-01009-0

**Published:** 2024-10-01

**Authors:** Akinkunmi Paul Okekunle, Mary Nicolaou, Manuela De Allegri, Karlijn A.C. Meeks, Hibbah Osei-Kwasi, Julia Stockemer, Ama de-Graft Aikins, Isaac Agbemafle, Silver Bahendeka, Daniel Boateng, Kerstin Klipstein-Grobusch, Erik Beune, Charles Agyemang, Matthias B. Schulze, Ina Danquah

**Affiliations:** 1 Heidelberg Institute of Global Health (HIGH), Medical Faculty and University Hospital, Heidelberg University, Heidelberg, Germany; 2 Department of Public & Occupational Health, Amsterdam UMC, Amsterdam Public Health Research Institute, University of Amsterdam, Meibergdreef 9, Amsterdam, 1105 AZ The Netherlands; 3 Center for Research on Genomics and Global Health, National Human Genome Research Institute, National Institutes of Health, Bethesda, MD USA; 4 Department of Clinical Sciences and Nutrition, University of Chester, Chester, UK; 5 Institute of Advanced Studies, University College London, London, UK; 6 Fred N. Binka School of Public Health, University of Health and Allied Sciences, Ho, Ghana; 7 Department of Internal Medicine, St. Francis Hospital Nsambya, MKPGMS-Uganda Martyrs University, Kampala, Uganda; 8 Julius Global Health, Department of Global Public Health and Bioethics, Julius Center for Health Sciences and Primary Care, University Medical Center Utrecht, Utrecht University, Utrecht, The Netherlands; 9 Division of Epidemiology and Biostatistics, School of Public Health, Faculty of Health Sciences, University of the Witwatersrand, Johannesburg, South Africa; 10 Department of Molecular Epidemiology, German Institute of Human Nutrition Potsdam-Rehbruecke, Nuthetal, Germany; 11 Institute of Nutritional Science, University of Potsdam, Nuthetal, Germany; 12 Hertz-Chair “Innovation for Planetary Health” at Transdisciplinary Research Area “Technology and Innovation for Sustainable Futures” and Center for Development Research (ZEF), Rheinische Friedrich-Wilhelms- University Bonn, Genscherallee 3, 53113 Bonn, Germany; 13 Department of Medicine, Johns Hopkins University School of Medicine, Baltimore, Maryland, USA

**Keywords:** Diet sustainability, Diet quality, Climate-friendliness, RODAM

## Abstract

**Background:**

The sustainability of diets consumed by African populations under socio-economic transition remains to be determined. This study developed and characterized a multi-dimensional Sustainable Diet Index (SDI) reflecting healthfulness, climate-friendliness, sociocultural benefits, and financial affordability using individual-level data of adults in rural and urban Ghana and Ghanaian migrants in Europe to identify the role of living environment in dietary sustainability.

**Methods:**

We used cross-sectional data from the multi-centre Research on Obesity and Diabetes among African Migrants Study (*N* = 3169; age range: 25–70 years). For the SDI construct (0–16 score points), we used the Diet Quality Index-International, food-related greenhouse gas emission, the ratio of natural to processed foods, and the proportion of food expenditure from income. In linear regression analyses, we estimated the adjusted ß-coefficients and 95% confidence intervals (CIs) for the differences in mean SDI across study sites (using rural Ghana as a reference), accounting for sociodemographic and lifestyle factors.

**Results:**

The overall mean SDI was 8.0 (95% CI: 7.9, 8.1). Participants in the highest SDI-quintile compared to lower quintiles were older, more often women, non-smokers, and alcohol abstainers. The highest mean SDI was seen in London (9.1; 95% CI: 8.9, 9.3), followed by rural Ghana (8.2; 95% CI: 8.0, 8.3), Amsterdam (7.9; 95% CI: 7.7, 8.1), Berlin (7.8; 95% CI: 7.6, 8.0), and urban Ghana (7.7; 95% CI: 7.5, 7.8). Compared to rural Ghana, the differences between study sites were attenuated after accounting for age, gender and energy intake. No further changes were observed after adjustment for lifestyle factors.

**Conclusion:**

The multi-dimensional SDI describes four dimensions of dietary sustainability in this Ghanaian population. Our findings suggest that living in Europe improved dietary sustainability, but the opposite seems true for urbanization in Ghana.

**Supplementary Information:**

The online version contains supplementary material available at 10.1186/s12937-024-01009-0.

## Introduction

Food production is responsible for > 70% of freshwater use, 30% of greenhouse gas emissions (GHGE), and 80% of deforestation worldwide [1, 2], necessitating the need for sustainable diets that meet the populations’ nutritional requirements without compromising environmental sustainability. The Food and Agriculture Organization (FAO) defined sustainable diets as nutritionally safe and environmentally friendly while being socially accepted and reasonably priced [3]. This definition requires holistic constructs of dietary sustainability to explore the importance of dietary practices on global warming [4] and the risk of nutrition-related diseases [5], while ascertaining socio-cultural appropriateness and financial affordability.

So far, the critical relationships between human health and the environment have been extensively examined [2, 4, 6, 7] and culminated in the operationalization of the EAT-Lancet planetary healthy diet as “a safe operating space” for environmentally friendly and healthy dietary choices [8]. In addition, more complex indices were developed to measure the various dimensions of dietary sustainability in diverse populations [9–12]. However, it is unclear how the multiple dimensions of dietary sustainability can be operationalized among sub-Saharan African populations under rapid economic transition, such as through urbanization on the subcontinent and migration to high-income countries. We propose to address the lack of climate change and health research from sub-Saharan Africa [13] and contribute to counteracting the climate injustice between the Global North, where most GHGs are emitted, and the Global South, where most climate change impacts occur [14].

Furthermore, describing the adherence to sustainable diets and its associated factors among sub-Saharan African populations may guide the just transition towards sustainable food systems with important co-benefits for nutrition and health. For Ghanaian populations living in rural Ghana, urban Ghana and first-generation migrants in Europe, we have previously identified distinct dietary patterns with partially unexpected, beneficial associations with type 2 diabetes and CVD risk [15–17]. These living environments represent the stages of rapid economic development and urbanization in Ghana, as well as migration to Europe. Here, we hypothesize that the observed dietary shifts in this population facilitate improvements in diet quality and associated health co-benefits at the expenses of environmental friendliness, affordability and socio-cultural appropriateness. These insights can inform contextualized diets that respect all components of sustainability [3].

Therefore, the overarching goal of this study was to develop and characterize a Sustainable Diet Index (SDI) among Ghanaian adults from the same geographic origin but living in different environmental contexts and identifying factors associated with adherence to this SDI. The specific objectives were (i) to construct an SDI that reflect healthfulness, environmental friendliness, sociocultural appropriateness, and financial affordability according to the FAO definition of sustainable diets [3], (ii) to describe SDI adherence of Ghanaian adults living in rural Ghana, urban Ghana, Amsterdam, London and Berlin, and (iii) to identify socio-demographic and lifestyle factors associated with SDI adherence across these study sites.

## Materials and methods

### Study design and population

The Research on Obesity and Diabetes among African Migrants (RODAM) Study was conducted as a multi-centre cross-sectional study among Ghanaian adults living in Ghana and Europe [18]. The respective ethics committees in Ghana and the three European countries (Netherlands, Germany and the United Kingdom) reviewed and approved the study protocols before data collection began in each country. Informed written consent was also obtained from each participant before enrollment in the study. Ghanaian adults (age range: 25–70 years) were recruited in Amsterdam (*n* = 1900), Berlin (*n* = 662), London (*n* = 1258), urban Ghana (Kumasi and Obuasi, *n* = 1619), and rural Ghana (Ashanti region, *n* = 946) between 2012 and 2015. The primary aim of the RODAM study was to characterize the genetic, environmental and behavioural risk factors of obesity and type 2 diabetes among Ghanaian populations under transition.

### Recruitment and data collection

The rationale, conceptual framework, study design, recruitment, and data collection strategies of the RODAM Study have been reported elsewhere [18–20]. Data collection was standardized across all study sites. In brief, trained personnel collected data through extensive general questionnaires, comprehensive assessment of dietary habits, physical examinations, and biological sample collection (fasting blood, urine). In each location, the questionnaires were administered in the participants’ preferred language (English, German, Dutch or Ghanaian language - i.e. Ewe, Akan, Ga) to elicit information on demographic, socioeconomic and lifestyle factors, among others.

Trained personnel conducted anthropometric examinations using validated devices and followed standard operating procedures. Body weight and height were measured (SECA 877 and SECA 217, Germany), and body mass index (BMI) was calculated as weight divided by height squared (kg/m^2^). The same assessor took anthropometric measurements twice, and the mean of the two measurements was used for analyses and classified according to the World Health Organization cut-off points [19].

**Fig. 1:**
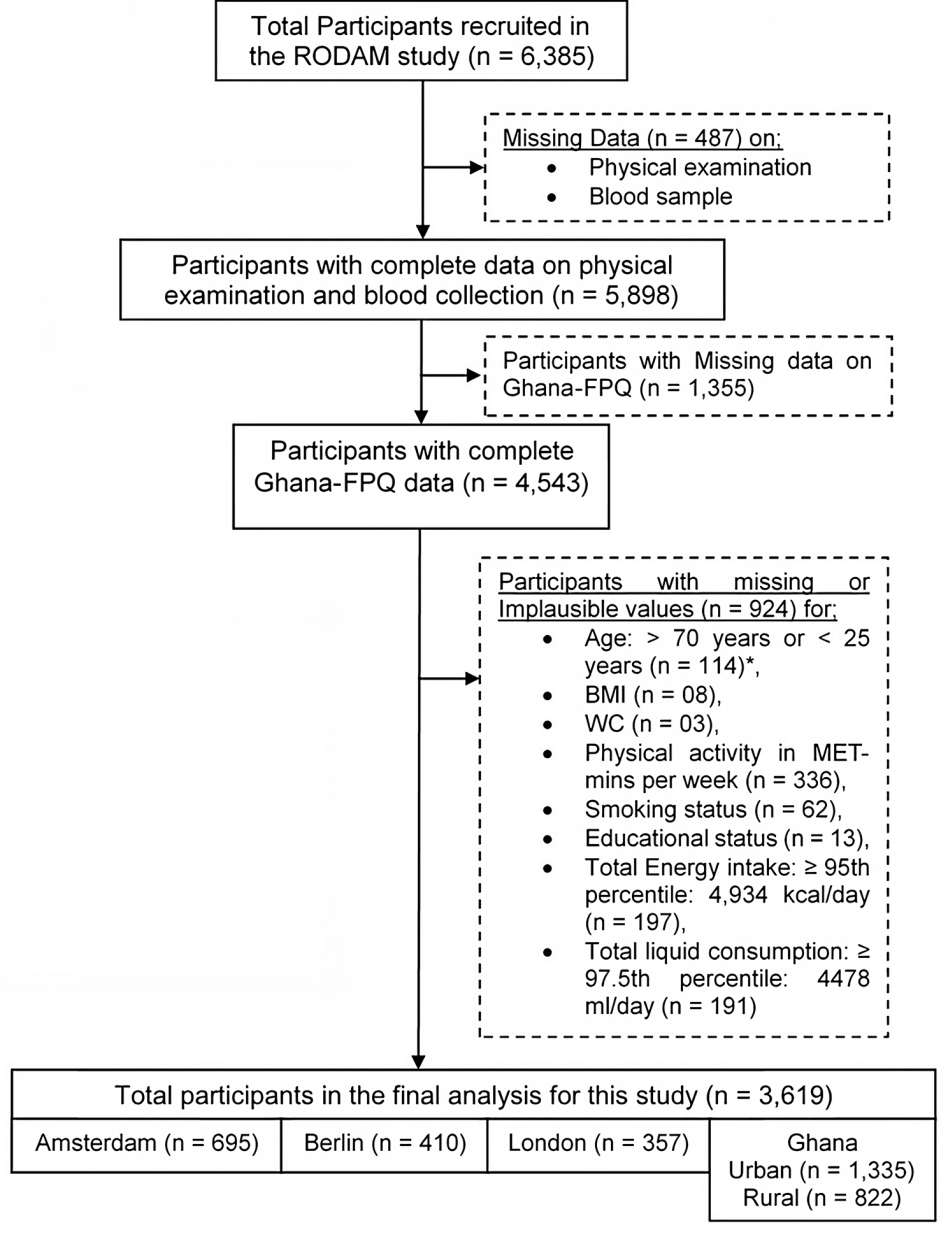
Flowchart describing how 3,619 Ghanaian adults were selected from the RODAM dataset for the current study. Participants were excluded as a result of missing or implausible data. FPQ: Food Propensity Questionnaire, BMI: body mass index, WC: waist circumference. *excluded to avoid probable or suspected implausible food intake among older and young population

Overall, 6,385 participants were recruited in the RODAM Study, with a 53–76% participation rate (Amsterdam – 53%, Berlin – 68%, London – 75%, urban Ghana – 74% and rural Ghana – 76%), and approximately 99% of the Ghanaian participants in Europe were first-generation migrants. The final sample in the present analysis included 3,619 participants after excluding individuals with implausible or missing values for any variables of interest (Fig. 1).

### Components of the Sustainable Diet Index (SDI)

#### Dietary assessment (healthfulness component)

Dietary information was assessed using a semi-quantitative Ghana-Specific Food Propensity Questionnaire (Ghana-FPQ). Details of the dietary assessment have been described elsewhere [20]. The Ghana-FPQ was designed to assess the usual individual dietary intake of 134 food items at predefined portions during the past 12 months. Furthermore, 24-hour dietary recalls were conducted in a random subsample of the participants to elicit comprehensive information on recipes, foods representative of specific food groups, and site-specific portion sizes. Food intakes were converted into energy and nutrient consumption by linking the Ghana-FPQ data with the West African Food Composition Table [21] and the German Nutrient Database [22].

The diet quality of the food consumed by participants in the RODAM dataset was operationalized using the Diet Quality Index-International (DQI-I). The DQI-I is a reputable tool for determining and monitoring diet quality [23]. DQI-I outlines diet quality under four major domains: variety (0–20 score points), adequacy (0–40 score points), moderation (0–30 score points) and balance (0–10 score points). The variety domain was defined as a measure of between- and within-food group varieties. The adequacy domain accounted for the adequate intake of healthy foods and essential nutrients. The moderation domain accounted for the magnitude of foods and nutrients that should be consumed in moderation due to their associations with chronic diseases. The balance domain examined the proportionality of energy sources and dietary fat composition. Details of the DQI-I method in the RODAM dataset have been reported elsewhere [16]. In this study, the DQI-I score (0-100) represents the nutritional sub-index of the SDI. Higher DQI-I reflected better healthfulness within the SDI.

#### Assessment of GHG emissions (environmental component)

A diet-related environmental impact assessment was carried out using the life-cycle assessment (LCA) for the environmental footprints of food production and consumption [24]. This study estimated the individual carbon dioxide-equivalents (CO_2_e) as the sum of all food-related GHG emissions [9]. Environmental footprints of 130 food items and ten dishes in the Ghana-FPQ were profiled. Site-specific GHG emissions (including methane and nitrous oxide) were calculated for all participants using the International Standard Organization (ISO) standards (14040 and 14044) on product LCA [25, 26] and ISO 14,067 on the carbon footprint of products [27] with the support of the Institute for Environment and Energy Research, Heidelberg, Germany [28]. CO_2_e were reported in g/day per kilogram of ready-to-eat foods, considering land-use changes (especially those caused by deforestation for agricultural purposes) using an attributive land-use change approach [29, 30]. Lower CO_2_e translated into better environmental sustainability within the SDI.

#### Assessment of sociocultural appropriateness (sociocultural component)

In this study, we operationalized the sociocultural component of sustainable diets according to Seconda et al. 2019 [9]. As a proxy, they proposed the proportions of ready-made foods in the daily diet. The authors reason that frequent consumption of ready-made foods reduces the cooking activities, which however, provide an opportunity for social exchange, preserve culinary heritage, and avoid standardisation of recipes [9]. Here, we classified foods as natural or processed using the criteria by the Food Standards Agency [31]. The ratio of natural foods to processed foods (RNtP) was computed for each participant to reflect the sociocultural component of the SDI. Higher RNtP translated into better sociocultural appropriateness within the SDI.

#### Assessment of financial affordability (economic component)

Financial affordability is an economy-related factor in dietary sustainability and postulates that food expenditure is likely subject to income in a particular setting [32]. A well-established approach to discerning affordability is weighing the food-related cost against the income per individual. This study explored external databases [33–37] for site-specific price collection of aggregated food items in the RODAM dataset (between 2013 and 2015). We used the agric-tool database of the European Commission [34, 35] to identify food price collection (in EURO) for Ghanaian participants in Europe. For Ghana, we used the 2013 to 2015 data on the national average monthly retail food prices collection (in Ghana Cedi) made available by the Ghanaian Ministry of Agriculture [37]. Where food price information was absent in both databases, we utilized the food price repositories of the United States Department of Agriculture [36, 38] or the FAO [39, 40] to collate food prices taking into account standard currency conversion rates [41, 42].

Site-specific average monthly income data was sourced from external databases and assigned to participants. Participants from the Europe were assigned income (in EURO) according to the European Union statistics on country-specific income, living conditions and community household panel surveys taking into account differences between men and women [43]. For Ghana, participants were assigned income (in Ghana Cedi) according to the Ghana Living Standards Survey 2014 [44]. Site-specific food-related monetary expenditure was computed as the sum of food costs from food item consumption (g/d) multiplied by the average site-specific price of the same food [9]. The economic component of the SDI was estimated as the ratio of total food-related monetary expenditure to the assigned site-specific income (RFtI). Lower RFtI values denoted higher financial affordability within the SDI.

#### Construction of the Sustainable Diet Index (SDI)

The SDI components and the allocation of score points for the SDI are shown in Supplementary Table S1. We allocated 0 to 4 score points for each component according to the population quintile cut-offs. For quintiles of the DQI-I, CO_2_e percentiles and the RNtP, score points were allotted in increasing order, i.e., individuals in the first quintile received zero points, while individuals in the fifth quintile received four points. For the quintiles of RFtI, score points were allocated in decreasing order. SDI was constructed as the sum score of the components, translating into score points ranging from 0 to 16. Pearson’s correlation coefficients and weighted κ coefficients were computed for the components and modified SDI (by removing a component one at a time) with a post hoc difference in means across SDI quintiles, accounting for multiple comparisons using the least significant difference. The content validity and sensitivity analyses of each domain in the SDI are in Supplementary Table S2.

#### Assessment of demographic, socio-economic, and lifestyle factors

Demographic factors in this study comprised gender (male or female) and age (in years, classified as < 40 years, 40–64 years and ≥ 65 years). Socio-economic factors captured the number of people in the household (≤ 5 or > 5), marital status (never married, currently married or formerly married), educational level (never/elementary, lower/intermediate or higher/university) and employment status (not employed or employed). Lifestyle factors included smoking status (never, ever), alcohol use (no, yes), and physical activity in MET-minutes/week (which included physical activity at work, while commuting, and in leisure time), classified into ‘low to moderate’ or ‘high’ according to the Global Physical Activities Questionnaire [45]. Also, migration-related factors were documented as the length of stay in Europe (< 10 years, ≥ 10 years) for Ghanaian adults living in Amsterdam, Berlin and London.

### Statistical analysis

For characterizing our study population, we constructed SDI-quintiles and assessed the distribution of demographic, socio-economic, and lifestyle factors across these categories using the chi-square test and one-way analysis of variance for categorical and continuous data, respectively. We calculated means with 95% confidence intervals (CI) for continuous variables and percentages for categorical data. Next, we presented the means and 95% CIs of the SDI and its components across the five study sites. Using linear regression analyses, we then calculated the differences in mean SDI (95% CIs) between study sites accounting for potential confounding factors of SDI adherence and using rural Ghana as the reference category. Three models were constructed: Model 1 adjusted for age, gender, and energy intake; Model 2: additionally adjusted for marital status, educational level and employment status; Model 3: additionally adjusted for smoking status, alcohol consumption, and physical activity. Finally, we also constructed Model 4 for the population living in Europe to estimate the contribution of the length of stay to SDI adherence. A two-tailed *p*-value of < 0.05 was considered to be significant. All analyses were conducted using SAS 9.4 software (SAS Institute Inc.).

## Results

### Characteristics of the study population according to SDI adherence

Table 1 displays the distributions of demographic, socio-economic, lifestyle and location-related factors across SDI-quintiles. The mean age was 46.1 years (95% CI: 45.8, 46.5), and 63.5% were women. Mean age and the proportion of women increased across quintiles of SDI adherence. Regarding socio-economic factors, the mean number of people in the household was 4.3 (95% CI: 4.2, 4.4), 62.6% were married, 37.9% had at least elementary education, and 20.6% were unemployed. There were no clear trends for these variables across SDI-quintiles. For lifestyle factors, the proportion of people who never smoked was 91.1%, and 83.0% did not consume alcoholic beverages. The mean physical activity was 6676 MET-minutes/week (95% CI: 6438, 6914) and mean energy intake was 2511 kcal/d (95% CI: 2484, 2537). The proportions of people who never smoked or did not consume alcohol were highest in the fifth SDI-quintile, while physical activity and energy intake were lowest in the fifth SDI-quintile. Finally, the mean length of stay in Europe was 16.8 years (95% CI: 16.5, 17.1) and evenly distributed across SDI-quintiles, but mean BMI was 26.7 kg/m^2^ (95% CI: 26.5, 26.9), with lower BMI values across higher SDI-quintiles.

**Table 1:** Sociodemographic and lifestyle characteristics of 3,619 Ghanaian adults across quintiles (Q) of the SDI in the RODAM Study

Characteristics	Q1 [2.00–5.00] (*n* = 601)	Q2 [6.00–7.00] (*n* = 887)	Q3 [8.00–8.00] (*n* = 616)	Q4 [9.00–10.00] (*n* = 965)	Q5 [11.00–16.00] (*n* = 550)
Site (%)					
*Amsterdam*	14.8	24.6	21.8	18.3	14.0
*Berlin*	11.2	13.0	12.3	10.8	8.7
*London*	4.7	6.3	9.7	11.0	19.5
*Ghana (urban)*	48.9	37.3	33.3	32.3	35.1
*Ghana (rural)*	20.4	18.8	22.9	27.6	22.7
Length of stay^†^	17.3 (15.9, 18.7)	16.3 (15.3, 17.3)	17.2 (16.0, 18.4)	16.4 (15.4, 17.4)	17.6 (16.3, 18.8)
*< 10years*	26.9	28.2	23.9	27.8	24.9
*≥ 10 years*	73.1	71.8	76.1	72.2	75.1
Sex (%)					
*Male*	40.1	37.5	36.0	32.6	38.2
*Female*	59.9	62.5	64.0	67.4	61.8
Age (years)^†^	42.3 (41.4, 43.2)	44.7 (43.9, 45.4)*	46.7 (45.8, 47.5)*	47.6 (46.9, 48.3)*	49.6 (48.7, 50.5)*
*< 40*	43.3	35.3	27.3	24.2	18.9
*40–64*	53.5	60.5	67.5	69.4	73.6
*≥ 65*	3.2	4.2	5.2	6.4	7.5
Number of people in the household^†^	4.5 (4.3, 4.7)	4.2 (4.1, 4.4)	4.2 (4.0, 4.4)	4.3 (4.1, 4.5)	4.3 (4.0, 4.5)
*≤ 5 (%)*	66.7	69.6	70.4	67.5	71.0
*> 5*	33.3	30.4	29.6	32.5	29.0
Marital Status (%)					
*Never married*	17.9	16.0	12.8	13.4	7.8
*Married*	65.2	61.7	64.1	60.3	63.6
*Formerly married*	16.9	22.3	23.1	26.3	28.6
Educational level (%)					
*Never/Elementary*	30.6	34.6	39.4	43.9	39.1
*Lower/Intermediate*	59.7	56.8	52.3	48.3	50.4
*Higher/University*	9.7	8.6	8.3	7.8	10.5
Employment status (%)					
*No*	16.3	22.2	21.9	21.2	19.9
*Yes*	83.7	77.8	78.1	78.8	80.1
Smoking status (%)					
*Never*	87.2	90.5	91.7	93.4	91.8
*Ever*	12.8	9.5	8.3	6.6	8.2
Current alcohol use (%)					
*No*	76.7	78.1	82.1	86.6	92.2
*Yes*	23.3	21.9	17.9	13.4	7.8
Physical activity (MET-minutes/week)^†^	7762.9 (7180.1, 8345.8)	6892.7 (6412.9, 7372.5)*	6526.2 (5950.4, 7107.9)*	6273.5 (5813.5, 6733.4)*	6010.9 (5401.6, 6620.2)*
*Low – Moderate*	40.1	46.6	48.4	49.1	49.5
*High*	59.9	53.4	51.6	50.9	50.5
Energy intake (cal/day)^†^	2973 (2912, 3035)	2677 (2626, 2727)*	2524 (2463, 2585)*	2289 (2241, 2338)*	2111 (2046, 2175)*
BMI (kg/m^2^)^†^	26.8 (26.4, 27.3)	27.0 (26.6, 27.4)	27.3 (25.9, 26.6)	26.2 (25.9, 26.6)*	26.4 (25.9, 26.8)*

†means and 95% confidence interval

*mean values were significantly different (at *p* < 0.05) compared to Q1

### SDI and its components across study sites

**Fig. 2:**
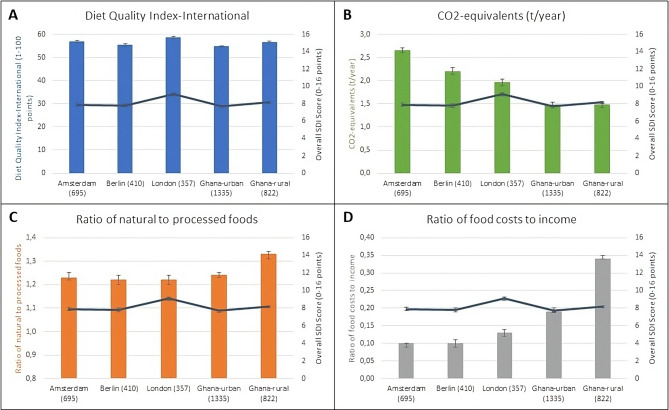
Means and 95% confidence interval (CI) of the Sustainable Diet Index (SDI) (dark blue line), and four components of the SDI among 3,619 Ghanaian adults **(A)** Diet Quality Index-International, **(B)** carbon equivalents (CO2e) in t/year, **(C)** ratio of natural to processed foods (RNtP), **(D)** Ratio of food costs to income (RFtI)

The distributions of the SDI, its components and the respective indicators across the study sites are presented in Fig. 2 and Supplementary Table S3. The SDI score ranged from 0 to 16, and the mean SDI in the study population was 8.0 (95% CI: 7.9, 8.1). This figure was highest in London, followed by rural Ghana, Amsterdam, Berlin and urban Ghana. The mean DQI-I was higher in rural areas than in urban settings within Ghana. Also, the mean CO_2_e was lower, and the mean ratio of natural to processed foods (RNtP) was higher in rural than urban Ghana. At the same time, the mean proportion of food expenses from income (RFtI) was almost double in rural Ghana than in urban Ghana (Fig. 2). Across the European sites, London showed the highest values for diet quality (mean DQI-I: 58.6; 95% CI: 57.9, 59.3) and GHG emissions (mean CO_2_e: 1965.2 t/year; 95% CI: 1892.0, 2038.4), while the food expenses in relation to income were highest in this location (mean RFtI: 0.13; 95% CI: 0.12, 0.14). Mean RNtP was similar in London, Amsterdam, and Berlin. Ghanaian migrants in Germany had the worst profile of SDI components: mean DQI-I was lowest, mean GHG emissions were highest, and mean RNtP was lowest. However, this group had the best financial affordability (RFtI) of the diet. Diet quality and GHG emissions were similar between Berlin and urban Ghana, but mean RNtP and mean RFtI were higher in urban Ghana than in Berlin.

### Factors associated with SDI adherence across study sites

Table 2 shows the contributions of demographic, socio-economic, and lifestyle factors to site-specific SDI adherence. In Ghana, the differences in mean SDI strengthened after adjustment for age, gender and energy intake, translating into a 0.04 lower SDI score in urban Ghana than in rural Ghana. Models 2 and 3 suggested no additional changes when considering socio-economic and lifestyle variables. For the differences between rural Ghana and European study sites, the various factors of adherence had distinct contributions: Lower mean SDI in Amsterdam as compared to rural Ghana was still discernible after adjustment for age, gender, energy intake, and socio-economic factors (Model 2), but this difference attenuated to the null when lifestyle factors were taken into account (Model 3). Also, the lower mean SDI in Berlin compared to rural Ghana was explained mainly by age, gender, and energy intake (Model 1), and the difference was no longer discernible in Models 2 and 3. Lastly, the higher mean SDI in London compared to rural Ghana remained robust, even after adjustment for all potential factors of adherence and was explained mainly by socio-economic variables (Table 2). We also considered the length of stay in Europe as a factor in SDI adherence (Table 3). This variable neither added to the explained variation in SDI nor changed the beta-coefficients.

**Table 2:** Adjusted associations of study site with SDI-adherence among 3,619 Ghanaian adults, using rural Ghana as the reference category

Model	ß coefficients	95% CI	*p*-value	Adjusted *R*^2^
**Amsterdam**				
Unadjusted	− 0.01	− 0.02, 0.00	0.018	0.00
Model 1	− 0.02	− 0.04, 0.01	< 0.001	0.03
Model 2	− 0.02	− 0.03, 0.01	0.003	0.16
Model 3	− 0.01	− 0.02, 0.00	0.100	0.23
**Berlin**				
Unadjusted	− 0.02	− 0.03, 0.00	0.008	0.01
Model 1	− 0.01	− 0.02, 0.01	0.381	0.04
Model 2	− 0.00	− 0.01, 0.01	0.512	0.16
Model 3	0.00	− 0.01, 0.01	0.462	0.22
**London**				
Unadjusted	0.04	0.02, 0.05	< 0.001	0.03
Model 1	0.04	0.03, 0.06	< 0.001	0.06
Model 2	0.04	0.03, 0.05	< 0.001	0.26
Model 3	0.04	0.03, 0.05	< 0.001	0.26
**Urban Ghana**				
Unadjusted	− 0.02	− 0.03, − 0.01	< 0.001	0.01
Model 1	− 0.04	− 0.05, − 0.03	< 0.001	0.10
Model 2	− 0.04	− 0.05, − 0.03	< 0.001	0.10
Model 3	− 0.04	− 0.05, − 0.03	< 0.001	0.11

Beta-coefficients (ß), 95% confidence intervals (CIs), *p*-values, and adjusted R-Squared (R^2^) were estimated using linear regression

Model 1: adjusted for age (years, continuous), gender (male, female), and energy intake (kcal/d, continuous)

Model 2: Model 1 + marital status (‘never married, married, formerly married), education (‘Never/Elementary and Lower/Intermediate, Higher/University), employment status (no, yes)

Model 3: Model 2 + smoking status (never, ever), alcohol consumption (no, yes), and physical activity (MET-minutes/week, continuous)

**Table 3:** Adjusted associations of study site with SDI-adherence among 1,462 Ghanaian migrants in Europe

Model	Amsterdam	Berlin					London			
		ß coefficients	95% CI	*p*-value	Adjusted *R*^2^		ß coefficients	95% CI	*p*-value	Adjusted *R*^2^
Unadjusted	Ref.	− 0.01	− 0.01, 0.01	0.459	0.00		0.05	0.04, 0.06	< 0.001	0.06
Model 1	Ref.	0.02	0.01, 0.04	0.001	0.11		0.07	0.06, 0.08	< 0.001	0.18
Model 2	Ref.	0.02	0.01, 0.04	0.001	0.12		0.06	0.05, 0.07	< 0.001	0.32
Model 3	Ref.	0.02	0.01, 0.04	0.001	0.12		0.05	0.04, 0.06	< 0.001	0.36
Model 4	Ref.	0.03	0.01, 0.04	0.000	0.13		0.05	0.04, 0.06	< 0.001	0.36

Beta-coefficients (ß), 95% confidence intervals (CIs), *p*-values, and adjusted R-Squared (R^2^) were calculated by linear regression

Model 1: adjusted for age (years, continuous), gender (male, female), and energy intake (kcal/d, continuous)

Model 2: Model 1 + marital status (‘*never married*, *Married*,* formerly Married*), education (‘*Never/Elementary and Lower/Intermediate*,* Higher/University*), employment status (*no*, yes)

Model 3: Model 2 + smoking status (never, ever), alcohol consumption (no, yes), and physical activity (MET-minutes/week, continuous)

Model 4: Model 3 + length of stay (years, continuous)

## Discussion

### Summary of main findings

This study constructed an SDI derived from equally weighted components of healthfulness, environmental friendliness, sociocultural appropriateness and economic affordability to operationalize individual-level dietary sustainability and identified the importance of the living environment among Ghanaians living in their country of origin and Europe. Participants in London and rural Ghana presented higher diet sustainability than those in urban Ghana, Amsterdam and Berlin. In London, higher scoring in the healthfulness and environmental components came at the expense of lower sociocultural appropriateness and moderately high costs for food. Also, higher environmental and sociocultural component scores in rural Ghana were accompanied by poor diet quality and high food costs. The adherence to SDI and its components in urban Ghana were similar to those among Ghanaian migrants. The most relevant contributors to differences in SDI between study sites were age, gender, and energy intake, whereas socio-economic and lifestyle factors did not significantly contribute to SDI differences.

### Validity and geographic distribution of the SDI

Multi-dimensional indices that reflect the complexity of dietary sustainability have been constructed in the Spanish SUN cohort [46] and the French NutriNet-Santé study [9]. The content validity of the SDI in the RODAM study (Pearson correlation coefficients between 0.81 and 0.82) was similar to the NutriNet-Santé cohort (Pearson correlation coefficients between 0.85 and 0.92). In the SUN cohort and the NutriNet-Santé cohort, higher SDI scores were associated with lower risks of developing chronic diseases, including cancers, cardiovascular disease and type 2 diabetes [9, 46]. Pending the completion of follow-up in the RODAM study and the identification of incident cases of type 2 diabetes, lower baseline BMI values in higher SDI-quintiles hint towards similar health co-benefits of environmentally friendly diets in this Ghanaian population. Still, the construct validity of the present SDI remains to be evaluated in the RODAM Study.

The differences in dietary sustainability across multiple locations in our study were expected but are complex in interpretation. It was observed that poor financial affordability strongly contributed to lower total scores of the SDI in rural and urban Ghana. This impact of income on adherence to healthy and environmentally friendly diets accords with previous reports on the consistently high prices for nutrient-rich and environmentally-friendly foods in the face of dwindling income worldwide [47]. In fact, the mean monthly income in Ghana ranged between € 270 to € 495 per capita during the time of the study [43], and participants in Ghana spent between 19% and 34% of their income on food, while this proportion ranged between 10% and 13% in Europe. Currently, 38% of the world’s population earn a meagre income and cannot afford healthy or environmentally friendly diets [48]. At the same time, the high proportion of fresh and unprocessed foods in the Ghanaian diets correlated with lower CO_2_e, particularly in rural settings. This might have outweighed the strong negative impacts of financial constraints on the overall SDI. By definition, fresh and local food products come along with lower GHG emissions due to reduced food processing and transportation [24]. In fact, of the economically active population in the Ashanti Region, Ghana, about 60% are engaged in agriculture, forestry and fishery. These subsistence farmers consume their primary agricultural yield and purchase only a small amount of other foods in the markets [49, 50]. Still, the highest values of the total SDI were seen in London due to the best balance of the four components, especially from improved diet quality. We have previously described the improved diet quality among Ghanaian migrants in Europe compared to their counterparts in Ghana. This included enhanced dietary diversity and inverse associations of modernized dietary patterns with type 2 diabetes and predicted 10-year risk of cardiovascular disease [15–17].

### Factors of SDI adherence

Age, gender and energy intake majorly accounted for the differences between study sites. This observation is similar to previous studies in France, Argentina, Spain and the Mediterranean Basin [9, 11, 51, 52]. The positive associations with advanced age may stem from conscious dietary choices in older age groups that support the secondary prevention of age-related chronic diseases, including more plant-based dietary practices with low environmental impacts [53, 54]. Also, there is evidence that older individuals have different attitudes towards food consumption than younger generations. They are more economically motivated for their dietary choices than younger consumers [55], which translates into higher readiness for reducing food waste, limiting red and processed meat, and prioritizing plant-based proteins [56]. The difference between men and women concerning environmentally-friendly behaviour has been termed the “eco-gender gap” [57]. Potential reasons for this situation comprise the fear of being seen as feminine when adopting environmentally friendly practices, eco-friendly media campaigns mainly targeting women, and the public sustainability discourse primarily led by women [57]. This eco-gender gap might even be bigger in sub-Saharan African societies [58]. Finally, energy intake is related to SDI through its association with GHG emissions. Excessive energy intake and demands have been linked to higher GHG emissions [59, 60], and energy-dense foods have a considerable environmental footprint [8]. Indeed, our previous work identified larger portion sizes for energy-dense foods among this Ghanaian population [15], and the reduction of portion sizes has already been a vital aspect of a dietary weight-loss program for them [61]. Taken together, these factors might be the underlying drivers of dietary sustainability. It is, therefore, essential to understand and address these individual determinants of dietary choices for the transition towards a more sustainable food system.

### Strengths and limitations

The findings of this study need to be interpreted on the background of its strengths and limitations. Even though SDI was conceptualized using the FAO definition and objectively assessed using equally weighted four SDI components, misclassification bias may have occurred. The sociocultural appropriateness for estimating dietary sustainability was limited to the weight of natural to processed food consumption and does not include other factors such as food choices, accessibility, local norms and cultural practices that were unconsidered prior to the design of the RODAM study. The affordability component of SDI was estimated within the context of food-related costs only and assigned individualized income, thereby delimiting income variability across sites. This approach was necessary due to limited data on participants’ income. Indirect food-related costs and a constellation of income-related factors were unaccounted for. Purchasing power is likely to vary between populations. Also, we could not distinguish between food purchased and food produced or gifted when estimating food cost. Causal associations cannot be inferred for factors associated with dietary sustainability. This study did not consider the proximal drivers of dietary sustainability, such as food environment, living conditions, and coping strategies for the migrant population. In the light of ancestry and food culture, the present study population is unique. Therefore, the transferability of our findings to other sub-Saharan African populations in similar transitional stages is likely but remains to be verified.

The strength of this study includes being the first epidemiological data from sub-Saharan African populations to construct a multi-dimensional SDI, which made it possible to study the significance of living environment (as a proxy for urbanization and migration) on climate friendliness. Our participants had a shared geographic origin and culture but lived in different places. This itemizes the importance of context-specific and culturally relevant strategies in promoting diet-related climate friendliness. The large sample size and the highly standardized data assessments across all sites are the unique strengths of this study. Country-specific LCA databases are germane in deriving CO_2_e data in understanding diet sustainability. Objective measures for evaluating income would be vital in methodological considerations for estimating financial affordability in purchasing power parities. Future diet assessment studies intending to estimate the significance of diet affordability should consider methodologies and validated instruments for collating data on food-related costs, practices, and ethos of obtaining food for consumption.

## Conclusion

In this study, we constructed an index to reflect the four dimensions of sustainable diets: healthfulness, climate-friendliness, sociocultural appropriateness, and financial affordability. This SDI allows the comparison of dietary sustainability among Ghanaian populations living in different environmental contexts. Living in Europe appears to exert beneficial effects on healthfulness and affordability but not on climate-friendliness and sociocultural appropriateness. Urbanization in Ghana seems to improve the affordability of diets, but it was characterized by poor diet quality, high GHG emissions, and loss of sociocultural appropriateness. The climate-friendliness of diets is highly context-specific, and it is pertinent for future interventions to consider these differences. Transformations towards sustainable diets need to address the impacts of age, gender roles, and portion sizes on dietary practices among this Ghanaian population under transitions.

## Electronic supplementary material

Below is the link to the electronic supplementary material.

Supplementary Material 1. Table S1: Description of indicators, components, point allocation and computation of components of the sustainable diet index (SDI) in the RODAM Study. Table S2: Mean and 95% confidence interval (CI), Pearson’s correlation, and weighted Kappa statistics for sensitivity analysis (by excluding a component at a time) across quintiles (Q) of the SDI in the RODAM Study. Table S3: Mean and 95% confidence interval (CI) of Sustainable diet index (SDI), components and indicators by site among 3,619 Ghanaian adults in the RODAM Study.

## Data Availability

The data presented in this study are available on request from the corresponding author.
