# Supplementary material for: A multi-dimensional Sustainable Diet Index (SDI) for Ghanaian adults under transition: the RODAM Study
Source: Nutr J. 2024 Oct 1;23:117. doi: 10.1186/s12937-024-01009-0 (PMC11443685; doi:10.1186/s12937-024-01009-0)
Supplement: Supplementary file 1 — Supplementary Material 1. Table S1: Description of indicators, components, point allocation and computation of components of the sustainable diet index (SDI) in the RODAM Study. Table S2: Mean and 95% confidence interval (CI), Pearson’s correlation, and weighted Kappa statistics for sensitivity analysis (by excluding a component at a time) across quintiles (Q) of the SDI in the RODAM Study. Table S3: Mean and 95% confidence interval (CI) of Sustainable diet index (SDI), components and indicators by site among 3,619 Ghanaian adults in the RODAM Study. [file 12937_2024_1009_MOESM1_ESM.docx]

| Table S1: Description of indicators, components, point allocation and computation of components of the sustainable diet index (SDI) in the RODAM Study | | | | | | |
| --- | --- | --- | --- | --- | --- | --- |
| SDI components | Indicators | Sub-indicators | Description | SDI | Score points | Point allocation |
| Nutritional | DQI-I | Variety: 0 – 20 pts | DQI-I was a composite measure of diet assessment that accounts for diet's healthfulness and nutrient adequacy. Higher DQI-I scores denoted an excellent level of healthfulness and nutrient adequacy and better sustainability. | Healthfulness and diet quality | 0-4 | 0pt - ≤ 50.00  1pt – 51.00 < DQI-I ≤ 54.00  2pt – 55.00 < DQI-I ≤ 58.00  3pt – 59.00 < DQI-I ≤ 61.00  4pt – DQI-I > 62.00 |
|  |  | Adequacy: 0 – 40 pts |  |  |  |  |
|  |  | Moderation: 0 – 30 pts |  |  |  |  |
|  |  | Balance: 0 – 10 pts |  |  |  |  |
|  |  | Overall DQI Score: 0–100 pts |  |  |  |  |
|  |  |  |  |  |  |  |
| Environmental | Carbon  Footprints (CO_2_e) | CO_2_e g/day | This component applied the LCA, a multifactor measure of food-related anthropogenic activity that affects the environment and leads to climate change, to estimate the carbon footprints of foods. They include greenhouse gas emissions, non-renewable energy-dependent food systems, etc. CO_2_e (g/day) was transformed to the CO_2_e percentile. Higher percentiles indicated lesser CO_2_e and consequently lower impact on the environment and biodiversity | Environmental impact, biodiversity and climate change | 0-4 | 0pt – ≤ 57.00  1pt – 58.00 < CO_2_e ≤ 68.00  2pt – 69.00 < CO_2_e ≤ 75.00  3pt – 76.00 < CO_2_e ≤ 81.00  4pt – CO_2_e > 82.00 |
|  |  | CO_2_e t/year |  |  |  |  |
|  |  | CO_2_e percentile: 0 – 100 pts |  |  |  |  |
|  |  |  |  |  |  |  |
| Sociocultural | RNtP | The ratio of consumption of natural foods to processed food products | This was designed to assess the consumption of natural foods to processed/ food products. Higher RNtP implied lower consumption of processed and higher sustainability | Availability and accessibility of diets promote the development | 0-4 | 0pt – ≤ 0.85  1pt – 0.86 < RNtP ≤ 1.44  2pt – 1.45 < RNtP ≤ 2.26  3pt – 2.27 < RNtP ≤ 3.47  4pt – RNtP > 3.51 |
|  |  |  |  |  |  |  |
| Economic | RFtI | The share of food-related costs with reference to income | This index assessed whether food costs were high relative to income. Higher RFtI indicated higher food costs, lesser affordability and less sustainability. | Affordability of the diet | 0-4 | 4pt – ≤ 0.077  3pt – 0.078 < RFtI ≤ 0.141  2pt – 0.142 < RFtI ≤ 0.209  1pt – 0.211 < RFtI ≤ 0.296  0pt – RFtI > 0.299 |
| Total | SDI |  | Nutritional (DQI-I) + environmental (CO_2_e) + economic (RNtP) + sociocultural (RFtI) |  | 0-16 |  |
| DQI-I: diet quality index international. CO2e – carbon dioxide emissions, RNtP: ratio of natural to processed and/foods, RFtI: ratio of food-related to income, LCA: life cycle assessment, Pts: points | | | | | | |

| Table S2: Mean and 95% confidence interval (CI), Pearson's correlation, and weighted Kappa statistics for sensitivity analysis (by excluding a component at a time) across quintiles (Q) of the SDI in the RODAM Study | | | | | | | | | | | | | | | | | | | |
| --- | --- | --- | --- | --- | --- | --- | --- | --- | --- | --- | --- | --- | --- | --- | --- | --- | --- | --- | --- |
|  | Q1 (810) | |  | Q2 (393) | |  | Q3 (974) | |  | Q4 (825) | |  | Q5 (617) | |  |  |  |  |  |
|  | Mean | 95% CI |  | Mean | 95% CI |  | Mean | 95% CI |  | Mean | 95% CI |  | Mean | 95% CI |  | *r** | 95% CI | κ^‡^ | 95% CI |
| Overall SDI score | 4.30 | 4.26, 4.35 |  | 6.54 | 6.50, 6.58 |  | 8.00 | 7.95, 8.04 |  | 9.47 | 9.43, 9.51 |  | 11.65 | 11.60, 11.70 |  |  |  |  |  |
|  |  |  |  |  |  |  |  |  |  |  |  |  |  |  |  |  |  |  |  |
| SDI score without DQI-I | 3.00 | 2.98, 3.12 |  | 4.82 | 4.73, 4.91 |  | 6.08 | 5.97, 6.19 |  | 7.28 | 7.19, 7.37 |  | 8.71 | 8.59, 8.82 |  | 0.81 | 0.80, 0.82 | 0.58 | 0.56, 0.59 |
|  |  |  |  |  |  |  |  |  |  |  |  |  |  |  |  |  |  |  |  |
| SDI score without CO_2_e | 3.93 | 3.84, 4.01 |  | 5.22 | 5.15, 5.29 |  | 5.94 | 5.86, 6.02 |  | 6.73 | 6.67, 6.80 |  | 8.24 | 8.15, 8.33 |  | 0.81 | 0.80, 0.82 | 0.56 | 0.54, 0.58 |
|  |  |  |  |  |  |  |  |  |  |  |  |  |  |  |  |  |  |  |  |
| SDI score without RNtP | 2.77 | 2.65, 2.88 |  | 4.93 | 4.83, 5.02 |  | 6.03 | 5.92, 6.14 |  | 7.19 | 7.10, 7.28 |  | 8.97 | 8.85, 9.09 |  | 0.82 | 0.81, 0.83 | 0.57 | 0.55, 0.58 |
|  |  |  |  |  |  |  |  |  |  |  |  |  |  |  |  |  |  |  |  |
| SDI score without RFtI | 3.20 | 3.09, 3.32 |  | 4.65 | 4.56, 4.74 |  | 5.93 | 5.82, 6.04 |  | 7.20 | 7.11, 7.29 |  | 9.03 | 8.91, 9.15 |  | 0.82 | 0.81, 0.83 | 0.58 | 0.56, 0.59 |
| *Pearson correlation coefficients  ‡ Weighted kappa coefficients  DQI-I: diet quality index international  CO_2_e; carbon dioxide emission percentiles  RNtP: the ratio of natural to processed foods consumed  RFtI: site-specific ratio of individual food costs to income in a month | | | | | | | | | | | | | | | | | | | |

| Table S3: Mean and 95% confidence interval (CI) of Sustainable diet index (SDI), components and indicators by site among 3,619 Ghanaian adults in the RODAM Study | | | | | | | | | | | | |
| --- | --- | --- | --- | --- | --- | --- | --- | --- | --- | --- | --- | --- |
|  | Amsterdam (695) | | Berlin (410) | | London (357) | | Ghana-urban (1335) | | Ghana-rural (822) | | Total (3619) | |
|  | Mean | 95% CI | Mean | 95% CI | Mean | 95% CI | Mean | 95% CI | Mean | 95% CI | Mean | 95% CI |
| **Overall SDI Score** | 7.9^†^ | 7.7, 8.1 | 7.8^†^ | 7.6, 8.0 | 9.1^†^ | 8.9, 9.3 | 7.7^†^ | 7.5, 7.8 | 8.2 | 8.00, 8.3 | 8.0 | 7.9, 8.1 |
|  |  |  |  |  |  |  |  |  |  |  |  |  |
| **Nutritional component** | |  |  |  |  |  |  |  |  |  |  |  |
| DQI-I, total (100) | 57.0 | 56.5, 57.5 | 55.4^†^ | 54.7, 56.0 | 58.6^†^ | 57.9, 59.3 | 54.8^†^ | 54.5, 55.2 | 56.7 | 56.2, 57.2 | 56.1 | 55.9, 56.3 |
| Variety (0-20) | 15.9^†^ | 15.6, 16.2 | 15.2 | 14.8, 15.6 | 17.1^†^ | 16.7, 17.5 | 15.7^†^ | 15.5, 15.9 | 15.1 | 14.8, 15.40 | 15.69 | 15.6, 15.8 |
| Adequacy (0-40) | 33.6^†^ | 33.1, 33.8 | 33.3^†^ | 33.0, 33.6 | 34.5^†^ | 34.2, 34.8 | 31.9^†^ | 31.8, 32.1 | 32.5 | 32.3, 32.73 | 32.79 | 32.69, 32.9 |
| Moderation (0-30) | 6.8^†^ | 6.5, 7.1 | 6.2^†^ | 5.9, 6.6 | 6.4^†^ | 6.0, 6.8 | 6.5^†^ | 6.3, 6.7 | 8.5 | 8.2, 8.8 | 7.0 | 6.8, 7.1 |
| Overall balance (0-10) | 0.66 | 0.55, 0.78 | 0.62 | 0.33, 0.53 | 0.60 | 0.44, 0.76 | 0.76^†^ | 0.67, 0.84 | 0.57 | 0.46, 0.68 | 0.67 | 0.61, 0.72 |
| **Environmental component** | |  |  |  |  |  |  |  |  |  |  |  |
| CO_2_e g/day | 4943.0^†^ | 4792.9, 5075.1 | 5098.3^†^ | 4914.7, 5281.9 | 4350.8 | 4154.0, 4547.6 | 5446.4^†^ | 5344.6, 5548.2 | 4500.8 | 4371.1, 4630.5 | 4985.7 | 4922.5, 5049.0 |
| CO_2_e t/year | 2660.2^†^ | 2607.7, 2712.6 | 2209.9^†^ | 2141.7, 2278.2 | 1965.2^†^ | 1892.0, 2038.4 | 1491.3 | 1453.4, 1529.1 | 1477.2 | 1428.9, 1525.4 | 1840.7 | 1813.1, 1868.3 |
| **Sociocultural component** | |  |  |  |  |  |  |  |  |  |  |  |
| RNtP | 1.23^†^ | 1.22, 1.25 | 1.22^†^ | 1.20, 1.24 | 1.22^†^ | 1.20, 1.24 | 1.24^†^ | 1.23, 1.25 | 1.33 | 1.31, 1.34 | 1.258 | 1.251, 1.265 |
| Natural foods (g/day) | 1461.5^†^ | 1419.1, 1503.9 | 1626.5^†^ | 1571.3, 1681.8 | 1617.4^†^ | 1558.2, 1676.5 | 1581.3^†^ | 1550.7, 1611.9 | 1891.4 | 1852.4, 1930.4 | 1637.4 | 1618.2, 1656.6 |
| Processed foods (g/day) | 1735.7^†^ | 1692.7, 1778.7 | 1690.2^†^ | 1634.2, 1746.3 | 1421.1^†^ | 1361.0, 1481.1 | 790.7^†^ | 759.7, 821.8 | 589.4 | 549.8, 628.9 | 1090.6 | 1066.2, 1114.9 |
| **Economic component** | |  |  |  |  |  |  |  |  |  |  |  |
| RFtI | 0.10^†^ | 0.09, 0.10 | 0.10^†^ | 0.09, 0.11 | 0.13^†^ | 0.12, 0.14 | 0.19^†^ | 0.19, 0.20 | 0.34 | 0.34, 0.35 | 0.197 | 0.192, 0.201 |
| Food cost^§^ (€) | 199.6^†^ | 190.6, 208.6 | 199.5^†^ | 187.7, 211.2 | 263.2 | 250.6, 275.8 | 97.9^†^ | 91.4, 104.5 | 94.3 | 85.9, 102.6 | 144.4 | 140.0, 148.9 |
| Income^§^ (€) | 1972.1^†^ | 1970.0, 1974.1 | 1928.1^†^ | 1925.5, 1930.2 | 1957.8^†^ | 1954.9, 1960.7 | 494.4^†^ | 492.9, 495.8 | 269.6 | 267.7, 271.5 | 1033.9 | 1009.0, 1058.8 |
| †: estimates significantly differed (at *P* < 0.0001) compared to Rural Ghana,  DQI-I: diet quality index International,  CO_2_: carbon dioxide,  RNtP: the ratio of natural to processed foods consumed,  RFtI: site-specific Ratio of total food costs to income in a month,  §: individual data was estimated in site-specific currencies (€ and ₵ for sites in Europe and Ghana, respectively) but converted and reported in € | | | | | | | | | | | | |
